# Supplementary material for: Nomilin from Yuzu Seed Has In Vitro Antioxidant Activity and Downregulates Melanogenesis in B16F10 Melanoma Cells through the PKA/CREB Signaling Pathway
Source: Antioxidants (Basel). 2022 Aug 23;11(9):1636. doi: 10.3390/antiox11091636 (PMC9495840; doi:10.3390/antiox11091636)
Supplement: Supplementary file 1 [file antioxidants-11-01636-s001.zip › antioxidants-1821947-supplementary.pdf]

Supplementary data

Nomilin Isolated from Yuzu Seed Shows *In Vitro* Antioxidant Activity and Downregulates Melanogenesis in B16F10 Melanoma Cells via PKA/CREB Signaling Pathway

Moon-Hee Choi [1], Seung-Hwa Yang [2], Nam Doo Kim [3] and Hyun-Jae Shin [1,2,*]

Table captions

Table S1 HPLC analysis conditions used for identification of limonoid aglycone and limonoid glucoside

Table S2 Phenolic compounds identified in limonoid aglycone extracts quantified by HPLC

Table S3 Phenolic compounds identified in limonoid glucoside extracts quantified by HPLC

Figure captions

Figure S1. ^{1}H-NMR spectrum of nomilin.

Figure S2. ^{13}C-NMR spectrum of nomilin.

Figure S3. Antioxidant activity profiles of LA1, LA2, LA3, LG1, LG2, and LG3. (a) DPPH radical scavenging activity and (b) ABTS radical scavenging activity.

Figure S4. HPLC chromatogram of the limonoid aglycone (LA1, LA2, LA3) and limonoid glucoside (LG1, LG2, LG3) using diode-array detection at 220 nm. (a) LA1; (b) LA2; (c) LA3; (d) standard mixture (1: limonin, 2: nomilin); (e) LG1; (f) LG2; (g) LG3; (h) standard mixture (1: chlorogenic acid, 2: caffeic acid, 3: rutin, 4: p-coumaric acid, 5: naringin, 6: hesperidin, 7: luteolin, 8: linoleic acid).

Supplementary Tables

Table S1 HPLC analysis conditions used for identification of limonoid aglycone and limonoid glucoside

	Limonid Aglycone	Limonoid Glucoside
Column	Shim-Pack GIS-ODS C18 column (4.6 x 250mm, 5μm)	Xbridge C18 (4.6 x 150 mm, 5μm)
Flow rate	0.7 mL/min	1.0 mL/min
Solvent A	Water (in 0.01% phosphoric acid)	Water (in 0.01% phosphoric acid)
Solvent B	Acetonitrile (in 0.01% phosphoric acid)	Methanol (in 0.01% phosphoric acid)
Mobile phase	0-40 min 20-60% B, 40-50 min 60-100% B, 50-60 min 100% B	0-50 min 20-70% B
Time	60 min	50 min
Temperature	30 ℃	R. T.
Detection wavelenght	210 nm	220 nm
Injection volume	20 μL	20 μL

Table S2 Phenolic compounds identified in limonoid aglycone extracts quantified by HPLC

No.	Standard	LA1	LA2	LA3
1	Limonin	641.4 mg/g	315.5 mg/g	595.1 mg/g
2	Nomilin	538.7 mg/g	690.7 mg/g	1725.8 mg/g

Table S3 Phenolic compounds identified in limonoid glucoside extracts quantified by HPLC

No.	Standard	LG1	LG2	LG3
1	Chlorogenic acid	409.35 mg/g	132.18 mg/g	8.34 mg/g
2	Caffeic acid	0.00 mg/g	0.00 mg/g	0.00 mg/g
3	Rutin	0.00 mg/g	142.22 mg/g	3.99 mg/g
4	p-Coumaric acid	39.24 mg/g	103.22 mg/g	5.96 mg/g
5	Naringin	430.17 mg/g	292.19 mg/g	48.87 mg/g
6	Hesperidin	436.76 mg/g	188.22 mg/g	7.52 mg/g
7	Luteolin	13.66 mg/g	28.61 mg/g	0.90 mg/g
8	Linoleic acid	3.52 mg/g	2.73 mg/g	34.65 mg/g

Supplementary Figures

Figure S1.

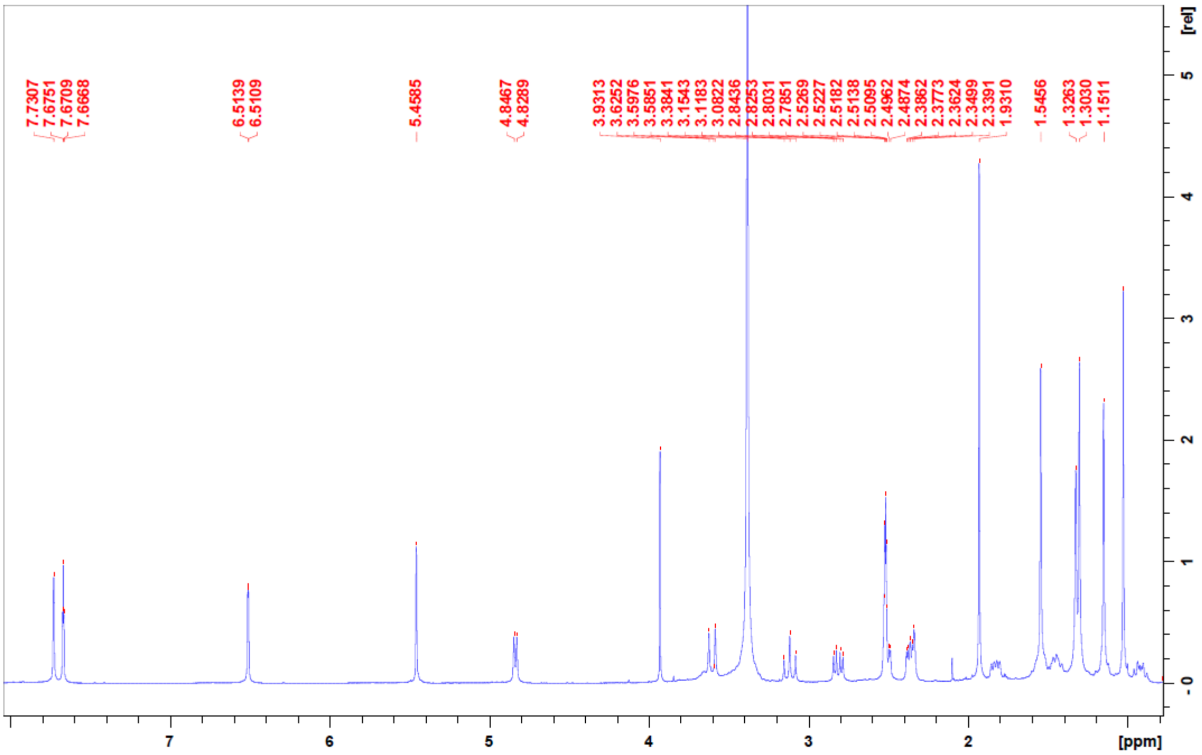

Figure S1. ^{1}H-NMR spectrum of nomilin.

Figure S2.

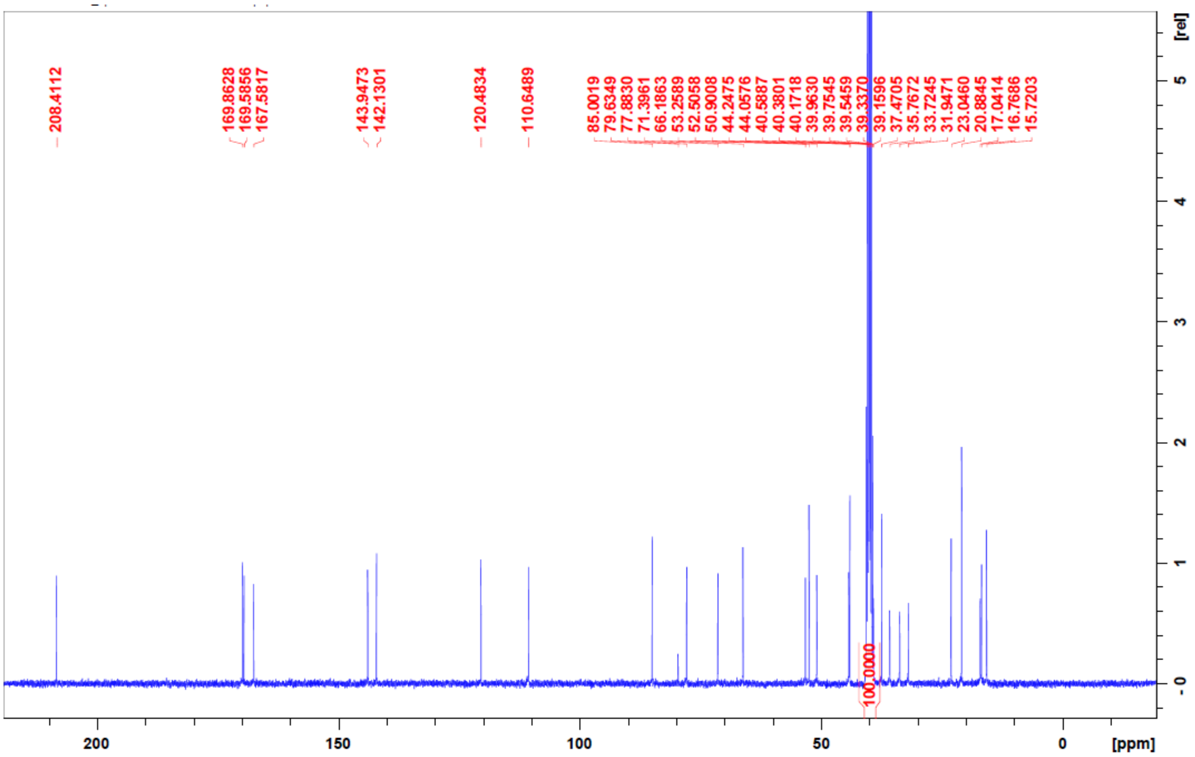

Figure S2. ^{13}C-NMR spectrum of nomilin.

Figure S3.

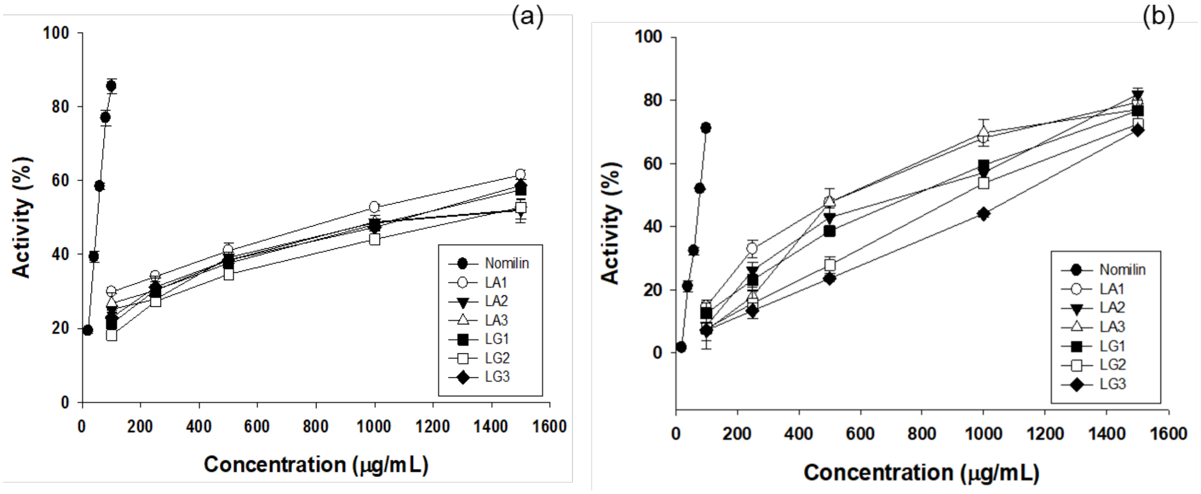

Figure S3. Antioxidant activity profiles of LA1, LA2, LA3, LG1, LG2, and LG3. (a) DPPH radical scavenging activity and (b) ABTS radical scavenging activity.

Figure S4.

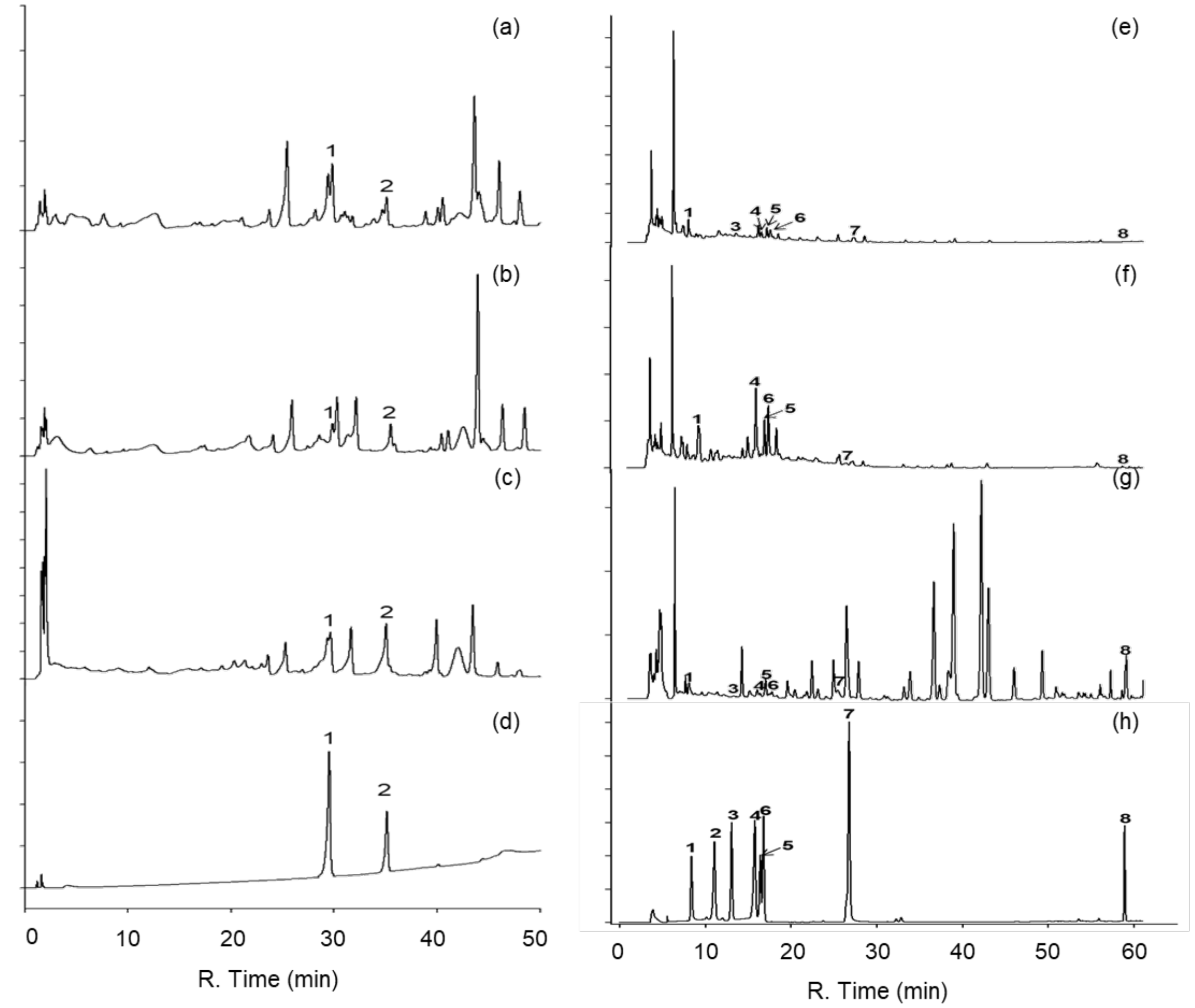

Figure S4. HPLC chromatogram of the limonoid aglycone (LA1, LA2, LA3) and limonoid glucoside (LG1, LG2, LG3) using diode-array detection at 220 nm. (a) LA1; (b) LA2; (c) LA3; (d) standard mixture (1: limonin, 2: nomilin); (e) LG1; (f) LG2; (g) LG3; (h) standard mixture (1: chlorogenic acid, 2: caffeic acid, 3: rutin, 4: p-coumaric acid, 5: naringin, 6: hesperidin, 7: luteolin, 8: linoleic acid).
